# 

**Published:** 1916-12-09

**Authors:** 


### The Travels of Scattered Hospital Staffs.

THE 1st Southern General Hospital, Birmingham, continues to improve the illustrations, which are now becoming perhaps the most successful feature in its journal, the *Southern Cross*. Apart from the drawings, which include a couple of cartoons, there are some interesting reproductions of photographs, showing men of the Midland Mountain Field Ambulance in Egypt. Such illustrations indicate how quickly the staff of these war hospitals may change, and how varied must be the experiences of the individuals who form their number. The life-history of these men, now mounted in apparent comfort on the hunches of camels, would probably reveal this photograph of them, so situated and appearing in a hospital gazette, as the most unexpected event in their lives. The editor, whose signature is N. Pollock (we will not risk prefix or pronoun here), should try to secure more of these photographs, so as to form a record of what the men of the 1st Southern did after they left, and where they went to. A subject suggestive also of articles.

### Hospital and Institutional Results in 1915.

**Manchester and Salford Lock Hospital.**—Amongst the out-patients treated last year, although the number of men fell slightly, that of women (1,732) was the highest in the four years for which statistics are given. The income was £822 and the expenditure £807.

**Livingstone College, Leyton.**—This college for training missionaries has been a temporary hospital for wounded soldiers since August 1915. There have been 850 admissions up to September 30, 1916; the income for hospital purposes in that period was £2,924 and the expenditure £2,761.

**Mildmay Medical Missions.**—The Charity includes the hospitals at Bethnal Green (fifty beds) and at Mildmay Park (thirty beds), and the mission with a dispensary at Old Ford. The accounts of each branch are kept separate, but the financial result of the Charity as a whole for 1915 showed that all expenditure had been met and there was a small balance in hand.

**Derbyshire Royal Infirmary.**—The accounts of this institution for some probably long-forgotten reason are made up from September 29 in one year to September 28 in another. The present, for reasons often expressed in these columns, is a good time for a Charity so placed to come into line with the vast majority of other charities, and we hope that in the near future we may congratulate the infirmary upon making this wise and necessary alteration. The out-patient and casualty attendances, contrary to general experience elsewhere, rose from 77,422 to 86,407 in succeeding periods of twelve months. The income for 1915-1916 was £19,902 and the expenditure £19,790.

### ORIGINAL RESEARCH AT THE DEVONSHIRE HOSPITAL, BUXTON.

THERE is every reason to hope that the Devonshire Hospital, Buxton, will soon succeed in clearing off the debt of £11,500 incurred through the addition of a modern thermal mineral-water bathing installation and other important improvements, as mentioned in THE HOSPITAL, December 2, 1916, p. 179. Last week Mr. W. Stevenson, the general superintendent and secretary, was handed three cheques of £1,000 each towards the liquidation of the deficit, contributed by Mr. R. Whitehead, Hargate Hall, Buxton; Mr. C. P. Markham, Ringwood, Chesterfield; and the directors of the Staveley Coal and Iron Company, Chesterfield.

RATES OF SUBSCRIPTION: Twelve months. 6/6; Six months, 4/-; Three months, 2/-; post free to any address in the United Kingdom. Abroad, Twelve months, 8/8; Six months, 5/-; Three months, 2/6, post free. THE HOSPITAL "Index" to Volume LX., April to September 1916, is now ready, price 2½d. per copy, post free. Address The Manager, "THE HOSPITAL," The Hospital Building, 28/29 Southampton Street, Strand, London, W.C.

